# Expanding the definition beyond surveillance criteria reveals a large burden of osteomyelitis caused by group B *Streptococcus* in the United States Veterans Health Administration

**DOI:** 10.1186/s12879-022-07238-0

**Published:** 2022-03-08

**Authors:** Tayoot Chengsupanimit, Taissa A. Bej, Brigid Wilson, Richard E. Banks, Sunah Song, Janet M. Briggs, Robin L. P. Jump, Federico Perez

**Affiliations:** 1 Department of Medicine, University Hospitals Cleveland Medical Center, Cleveland, OH USA; 2 Geriatric Research Education and Clinical Center (GRECC) 111O (W), VA Northeast Ohio Healthcare System, 10701 East Blvd., Cleveland, OH 44106 USA; 3 Division of Infectious Diseases and HIV Medicine in the Department of Medicine, Case Western Reserve University School of Medicine, Cleveland, OH USA; 4 Cleveland Institute for Computational Biology, Cleveland, OH USA; 5 Population and Quantitative Health Sciences, Case Western Reserve University School of Medicine, Cleveland, OH USA; 6 Case Western Reserve University -Cleveland VAMC Center for Antimicrobial Resistance and Epidemiology (Case VA CARES), Cleveland, OH USA

**Keywords:** *Streptococcus agalactiae*, Diabetes mellitus, Osteomyelitis

## Abstract

**Background:**

Population-based surveillance studies may underestimate osteomyelitis caused by Group B *Streptococcus* (GBS). We analyzed cases of GBS osteomyelitis, including patients diagnosed using an expanded case definition that incorporates cultures from non-sterile sites, as well as cultures from normally sterile sites.

**Methods:**

We retrospectively examined a cohort of veterans with the diagnosis of osteomyelitis between 2008 and 2017. Cases of *definite GBS osteomyelitis* required GBS isolation from normally sterile sites, (*e.g.*, blood or bone). Cases of *probable GBS osteomyelitis* permitted GBS isolation from non-sterile sites (*e.g.*, surgical sites, wounds). We compared comorbid conditions, lower extremity amputation and mortality rates in these groups.

**Results:**

Among 1281 cases of GBS osteomyelitis, the median age was 63 years, 87% had diabetes mellitus and 37% had peripheral vascular disease. Similar characteristics were found in 768 (60%) cases classified as definite and 513 (40%) classified as probable GBS osteomyelitis. Polymicrobial infection was less frequent in patients with definite than with probable GBS osteomyelitis (45% *vs.* 85%; P < 0.001). Mortality rates within 1-year were similar for definite and probable GBS osteomyelitis (12% *vs*. 10%). Amputation within 1-year occurred in 21% of those with definite and 10% of those with probable GBS osteomyelitis of the lower extremity, with comparable rates in the subset with monomicrobial infection.

**Conclusions:**

Expanding the definition of GBS osteomyelitis to include cases with cultures from non-sterile sites may be warranted, increasing the estimated burden of GBS osteomyelitis. This can help guide preventive efforts to reduce the impact of GBS osteomyelitis.

**Supplementary Information:**

The online version contains supplementary material available at 10.1186/s12879-022-07238-0.

## Introduction

Since the early 1940s, Group B *Streptococcus* (GBS) has been recognized as a clinically important and common cause of bacteremia and other infections in non-pregnant adults [1]. Population-based surveillance efforts document the emergence of invasive GBS infection as a cause of morbidity and mortality among older adults and among those with underlying medical conditions. Between 1990 and 2016, the incidence of invasive GBS infections among non-pregnant adults in the United Stated increased from 3.6 to 10.9 cases per 100,000 persons [2, 3]. Among adults, salient risk factors for invasive GBS infections include age ≥ 65, obesity, and diabetes mellitus [4, 5].

Osteomyelitis is among the most common forms of invasive GBS infection, particularly among individuals with diabetes mellitus [3, 5]. Case definitions utilized by the US Centers for Disease Control and Prevention’s (CDC) Active Bacterial Core Surveillance System to assess the incidence of invasive GBS infections, including osteomyelitis, require the isolation of GBS from a normally sterile site such as blood or bone [3]. Clinically, however, the diagnosis of osteomyelitis may be more complex and requires a combination of imaging, laboratory tests, histology, and clinical assessment accompanied by cultures from an anatomically relevant location, including non-sterile sites such as wounds [6, 7]. This is especially true of osteomyelitis in the setting of diabetic foot infection, which is due to contiguous infection from chronic wounds in patients with neuropathy and vascular insufficiency. These infections often are polymicrobial and determining the respective pathogenic role of GBS and other bacteria isolated from cultures is challenging. Diabetic foot osteomyelitis is a serious infection that frequently leads to amputation and is associated with a high risk of mortality [8, 9]. The requirement of isolating GBS only from cultures of normally sterile sites may underestimate the burden of osteomyelitis caused by this pathogen.

To better understand the epidemiology of GBS osteomyelitis, including cases diagnosed using cultures from non-sterile sites, we conducted a retrospective study using a cohort of patients who received care in hospitals from the US Veterans Health Administration (VHA) between 2008 and 2017. We hypothesized that patients diagnosed with GBS osteomyelitis using an expanded definition that included cultures from non-sterile sites would have similar clinical characteristics and outcomes compared to those diagnosed using the definition specific for invasive GBS infection that only recognizes cultures from sterile sites.

## Methods

### Study design and data sources

We conducted a retrospective cohort study of patients treated through the US Veterans Affairs (VA) healthcare system from January 1, 2008 through December 31, 2017, using the Veterans Affairs Informatics and Computing Infrastructure (VINCI) to access the VHA’s Corporate Data Warehouse (CDW). We used International Classification of Disease (ICD) codes to identify patients with a diagnosis of osteomyelitis and current procedural terminology (CPT) codes to select patients with imaging studies used to assess for osteomyelitis (Additional file 1: Tables S1, S2). Cultures were identified by searching microbiology tables for bacterial names containing the terms “Group B” and “agalactiae”; accompanying data fields described the anatomical source of the cultures. For patients who had ICD codes indicating lower extremity osteomyelitis, we also used CPT codes to identify patients who had a subsequent below- or above-knee amputation and the date of that surgery (Additional file 1: Tables S3, S4). The following data were also obtained from the CDW: patient demographics, height, weight, laboratory values, chronic medical conditions (based on ICD codes), and date of death. The Institutional Review Board (IRB) at the VA Northeast Ohio Healthcare System approved the study protocol.

### Case ascertainment and clinical characteristics

Inclusion criteria were VA healthcare users with an osteomyelitis diagnosis that coincided with a culture where GBS was isolated. The ICD code for osteomyelitis had to be entered within the 7 days before or the 30 days after the positive culture. Similar to previously described criteria, a case of *definite osteomyelitis* required the culture to come from a normally sterile site, specifically bone and/or blood cultures [3, 10]. A case of *probable osteomyelitis* permitted cultures collected from non-sterile sites, including deep tissue cultures or samples collected during surgical debridement, that were anatomically concordant with the location of the osteomyelitis [10]. We also required a case of *probable osteomyelitis* to have an imaging study used to assess for osteomyelitis (*i.e.*, radiography, computed tomography, nuclear, or magnetic resonance imaging) within 2 weeks of the diagnosis. For a given patient, only the first case of GBS osteomyelitis (incident case) during the 10-year study period was considered. Exclusion criteria were cases with an ICD code for osteomyelitis and a culture positive for GBS from a non-concordant anatomic site (*e.g.*, sputum or urine).

We evaluated the following patient characteristics: age, gender, race and ethnicity, comorbid conditions and Charlson comorbidity index (CCI), body mass index (BMI), and the percentage of glycated hemoglobin or hemoglobin A1c (HbA1c). To define monomicrobial (only GBS) and polymicrobial infections, we noted the presence of additional bacteria isolated from the same cultures that grew GBS, with specific attention to *Staphylococcus aureus* and *Pseudomonas aeruginosa*. The CCI was determined using ICD codes [11]. The presence of additional bacteria, BMI and HbA1c were determined as previously described [5]. The outcomes of interest were all-cause mortality at 30 days and 1 year, and, for the subset of patients with lower-extremity osteomyelitis, time to below- or above-knee amputation.

### Statistical analysis

Clinical and demographic characteristics of case groups were compared using t-tests for continuous variables and Chi-square tests for categorical variables. Kaplan–Meier curves for the year following the incident infection were used to estimate survival and, for those with lower extremity GBS osteomyelitis, time to amputation. Comparisons were made using a log-rank test. All statistical analyses were performed using R version 3.5.1 (R Project for Statistical Computing) including functions from the ICD and survival packages [12, 13]. All reported P-values are unadjusted.

## Results

Between 2008 and 2017, we identified 1281 patients with an incident case of GBS osteomyelitis. Of these, 768 (60%) cases were classified as definite GBS osteomyelitis and 513 (40%) as probable GBS osteomyelitis. The cohort consisted primarily of men (98%), with a mean age of 63.2 years (± 10.1) (Table 1). The most common medical condition identified was diabetes mellitus (87%) followed by peripheral vascular disease (37%) and heart disease (31%). A BMI ≥ 30, categorized as obese, was present in 51% of the cohort. The overall burden of comorbidities was lower among patients with definite osteomyelitis (CCI of 3.82 (± 2.3) compared to those with probable osteomyelitis [CCI of 4.52 (± 2.4); P < 0.001]. A HbA1C of ≥ 7.5% (exceeding the target of HbA1C 6.5%-7% for well controlled diabetes mellitus) was significantly more frequent among patients with definite compared to probable GBS osteomyelitis (72% *vs.* 53%, respectively; P < 0.001).

**Table 1:** Characteristics of patients from the US Veterans Health Administration with diagnosis of GBS osteomyelitis

Characteristics	All (n = 1281)	Definite GBS osteomyelitis (n = 768)	Probable GBS osteomyelitis (n = 513)	P-value^a^
Male sex, No. (%)^b^	1259 (98%)	756 (98%)	503 (98%)	0.762
Age, mean (± SD)^c^	63.2 (± 10.1)	62.8 (± 9.7)	63.7 (± 10.7)	0.127
Race				
White	926 (72%)	540 (70%)	386 (75%)	0.087
Black	253 (20%)	158 (21%)	95 (19%)	
Other^d^	101 (8%)	69 (9%)	32 (6%)	
Ethnicity				
Non-Latinx	1138 (89%)	673 (88%)	465 (91%)	0.054
Latinx	103 (8%)	73 (10%)	30 (6%)	
Other^d^	40 (3%)	22 (3%)	18 (4%)	
Charlson Comorbidity Index, mean (± SD)^c^	4.10 (± 2.3)	3.82 (± 2.3)	4.52 (± 2.4)	< 0.001
Specific medical comorbid conditions				
Diabetes mellitus	1120 (87%)	690 (90%)	430 (84%)	0.002
Peripheral vascular disease	468 (37%)	239 (31%)	229 (45%)	< 0.001
Heart disease	394 (31%)	227 (30%)	167 (33%)	0.282
Renal disease	392 (31%)	224 (29%)	168 (33%)	0.193
Pulmonary disease	340 (27%)	163 (21%)	177 (35%)	< 0.001
Stroke	192 (15%)	93 (12%)	99 (19%)	0.001
Cancer	157 (12%)	99 (13%)	58 (11%)	0.447
Liver disease	148 (12%)	68 (9%)	80 (16%)	0.001
AIDS/HIV	10 (1%)	7 (1%)	3 (1%)	0.566
Body mass index (BMI) ≥ 30	651 (51%)	382 (50%)	269 (52%)	0.374
Diabetic with a hemoglobin A1c ≥ 7.5	828 (65%)	554 (72%)	274 (53%)	< 0.001
Polymicrobial cultures^e^	779 (61%)	342 (45%)	437 (85%)	< 0.001
* Staphylococcus aureus*	418 (33%)	169 (22%)	249 (49%)	< 0.001
* Pseudomonas aeruginosa*	81 (6%)	27 (4%)	54 (11%)	0.005
Other organisms^f^	362 (28%)	182 (24%)	180 (35%)	0.607

^a^Compares definitive *vs.* probable osteomyelitis

^b^All values written as No. (%) unless otherwise indicated

^c^SD, standard deviation

^d^For Race includes American Indian, Alaska Native, Asian, Native Hawaiian or Pacific Islander and unknown; for Ethnicity includes unknown

^e^Cases could be included in more than one category, *i.e.,* a microbiology report that included GBS, *S. aureus*, and any other organisms would be included in the *S. aureus* as well as the “Other organism” row

^f^Includes potential pathogens, organisms not typically implicated in osteomyelitis (*i.e.*, yeast, *coagulase negative Staphylococci* spp.), and organisms that cultured and reported without further identification (*i.e.*, Gram-positive cocci or Gram-negative rods)

**Fig. 1:**
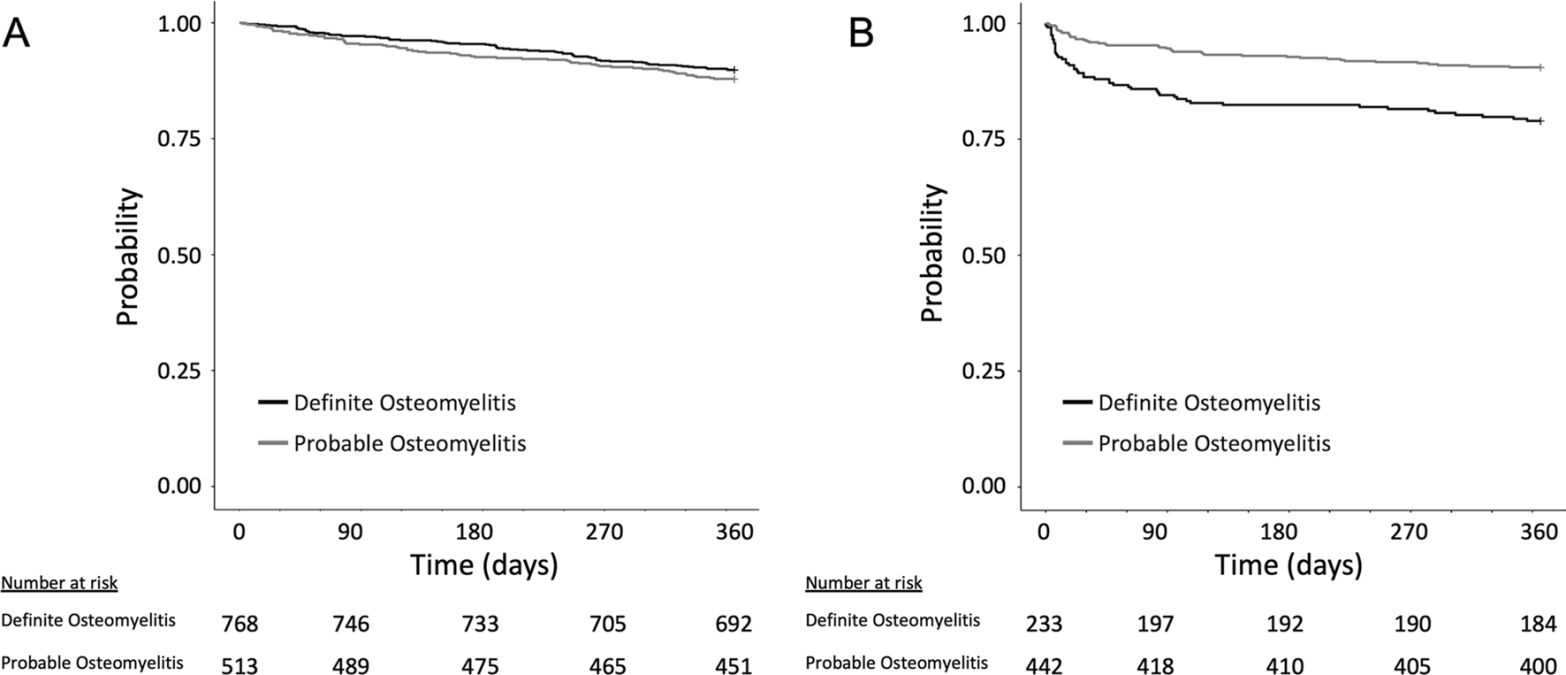
Kaplan–Meier curves of cases with definite (black) compared to probable (grey) GBS osteomyelitis. **A** Probability of survival at 1 year. **B** For those with lower extremity osteomyelitis, probability of avoiding a below- or above-knee amputation at 1 year

Polymicrobial infection (GBS and ≥ 1 additional bacteria) was less common among those with definite osteomyelitis (45%, 342/768) compared to probable osteomyelitis (85%, 437/513; P < 0.001). *Staphylococcus aureus* was the most common co-pathogen described, present in 22% and 49% for definite and probable osteomyelitis cases, respectively. All-cause mortality was similar at 30-days (3% and 5%, respectively) and 1-year (10% and 12%, respectively; log rank P = 0.26) for patients with definite and probable osteomyelitis (Fig. 1A). Among the 675 individuals with osteomyelitis of the lower extremity, the risk of progression to a below- or above-knee amputation at 1 year was 21% (49/233) compared to 10% (42/442; P < 0.01) for definite compared to probable GBS osteomyelitis (Fig. 1B).

**Fig. 2:**
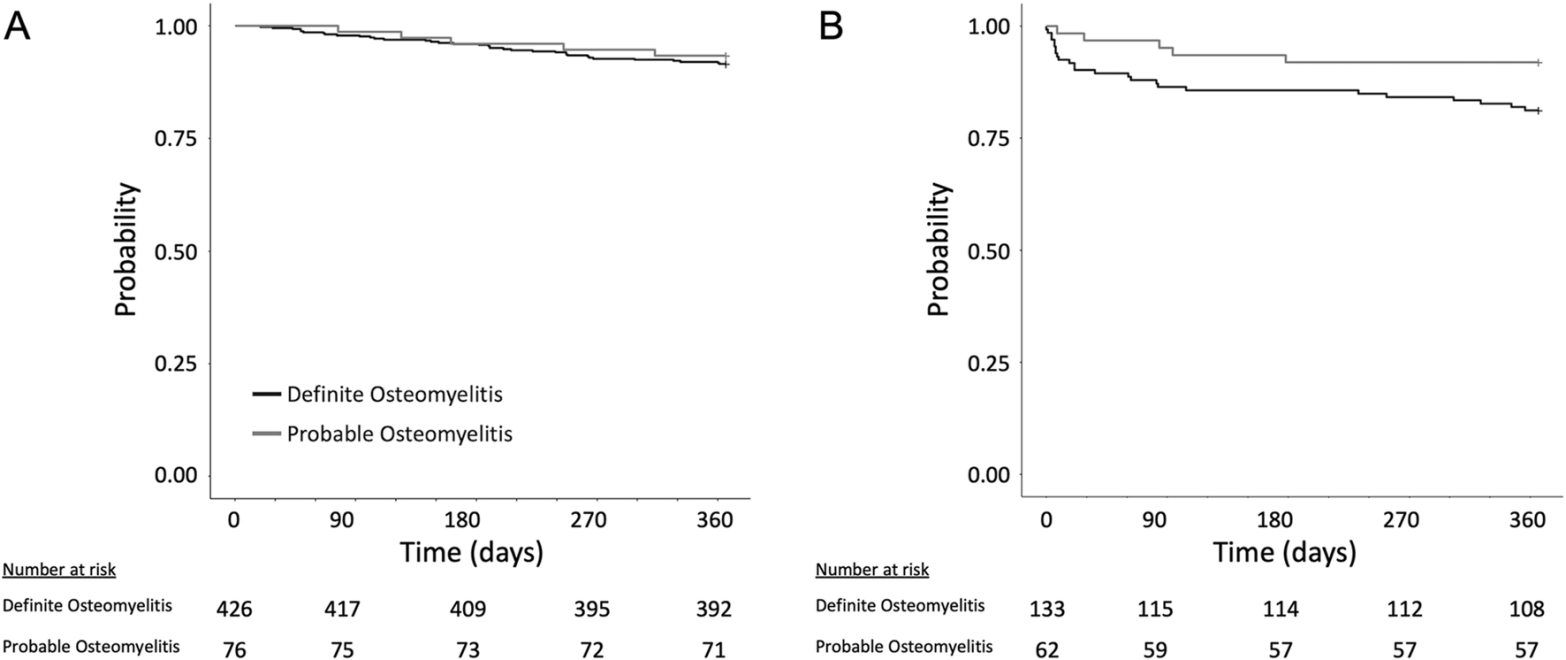
Kaplan–Meier curves of patients with definite (black) compared to probable (grey) GBS osteomyelitis, limited to those with cultures that were monomicrobial for GBS. **A** Probability of survival at 1 year. **B** For those with lower extremity osteomyelitis, probability of avoiding a below- or above-knee amputation at 1 year

In order to better assess the role of GBS as a pathogen in osteomyelitis, we analyzed the subset of cases with cultures that only grew GBS. The subset of patients with monomicrobial osteomyelitis due to GBS represented 39% (502/1281) of cases from the larger cohort. Similar to the larger cohort, the overall burden of comorbid conditions was lower among patients with definite compared to those with probable osteomyelitis (CCI of 3.82 ± 2.2 *vs.* 4.46 ± 2.6, respectively; P < 0.05). Both peripheral vascular disease (33% *vs.* 49%; P < 0.05) and chronic lung disease (23% *vs.* 37%, P < 0.05) were less common among those with definite compared to probable osteomyelitis (Table 2). All-cause mortality at 1 year among patients with cultures that were monomicrobial for GBS was 8% (36/426) and 7% (5/76; P = 0.58), respectively for those with definite compared to probable osteomyelitis (Fig. 2A). Analysis of lower-extremity cases with monomicrobial GBS infection revealed similar event rates to those observed overall, with a risk of a below- or above-knee amputation at 1 year of 19% (25/133) and 8% (5/62; P = 0.05) for patients with definite compared to probable GBS osteomyelitis (Fig. 2B).

**Table 2:** Characteristics of patients from the US Veterans Health Administration with monomicrobial GBS osteomyelitis

Characteristics	All (n = 502)	Definite GBS osteomyelitis (n = 426)	Probable GBS osteomyelitis (n = 76)	P-value^a^
Male sex, No. (%)^b^	493 (98%)	420 (99%)	73 (96%)	0.286
Age, mean (± SD)^c^	62.7 ± 9.6	62.7 ± 9.7	62.9 ± 9.1	0.833
Race				
White	353 (70%)	295 (69%)	58 (76%)	0.389
Black	101 (20%)	90 (21%)	11 (14%)	
Other^d^	48 (10%)	41 (10%)	7 (9%)	
Ethnicity				
Non-Latinx	431 (86%)	365 (86%)	66 (87%)	0.125
Latinx	54 (11%)	49 (12%)	5 (7%)	
Other^d^	17 (3%)	12 (3%)	5 (7%)	
Charlson Comorbidity Index, mean (± SD)^c^	3.92 ± 2.3	3.82 ± 2.2	4.46 ± 2.6	0.047
Specific medical comorbid conditions				
Diabetes mellitus	450 (90%)	387 (91%)	63 (83%)	0.057
Peripheral vascular disease	177 (35%)	140 (33%)	37 (49%)	0.011
Heart disease	143 (28%)	125 (29%)	18 (24%)	0.385
Renal disease	144 (29%)	118 (28%)	26 (34%)	0.309
Pulmonary disease	126 (25%)	98 (23%)	28 (37%)	0.016
Stroke	65 (13%)	50 (12%)	15 (20%)	0.084
Cancer	59 (12%)	52 (12%)	7 (9%)	0.580
Liver disease	57 (11%)	49 (12%)	8 (11%)	0.960
AIDS/HIV	5 (1%)	4 (1%)	1 (1%)	1.000
Body mass index (BMI) ≥ 30	246 (49%)	208 (49%)	38 (50%)	0.949
Diabetic with a hemoglobin A1c ≥ 7.5	356 (71%)	310 (73%)	46 (61%)	0.064

^a^Compares definitive *vs.* probable osteomyelitis

^b^All values written as No. (%) unless otherwise indicated

^c^SD, standard deviation

^d^For Race includes American Indian, Alaska Native, Asian, Native Hawaiian or Pacific Islander and unknown; for Ethnicity includes unknown

## Discussion

In this national cohort of patients from the VA, we analyzed more than 1250 cases of osteomyelitis caused by GBS. Of these, 40% were identified using an expanded case definition that included a diagnostic code for osteomyelitis and isolation of GBS from cultures of anatomically relevant non-sterile sites. These cases would not have been captured by case definitions conventionally used to conduct population-based surveillance of invasive GBS disease, including GBS osteomyelitis. By incorporating cases identified through our expanded criteria for osteomyelitis that included GBS recovered from non-sterile sites, we demonstrated that the burden of disease caused by GBS osteomyelitis appears to be greater than previously recognized.

As reported by both the US CDC as well as in our previous assessment of a cohort of US Veterans, osteomyelitis is among the most common invasive infections caused by GBS among non-pregnant adults [3, 5]. A study of 3 large counties in California found that, between 1995 and 2021, the rate of GBS osteomyelitis increased from 0.7 to 2.4 cases per 100,000 population [14]. These studies all used well-established definitions of invasive disease, requiring either positive bone or blood cultures to diagnose osteomyelitis. In clinical practice, however, the diagnosis of osteomyelitis may require serologic, imaging, and microbiological investigations, all of which need to be interpreted within the context of individual patients. For this study, we applied less stringent microbiological criteria, coupled with diagnostic and procedure (diagnostic imaging) codes available from clinical and administrative databases, to allow for recognition of probable GBS osteomyelitis. These aligned with criteria used by Kremers et al*.* in their description of the epidemiology of osteomyelitis in Olmsted County, Minnesota, in which they characterized cases as possible, probable and definite osteomyelitis based on the availability of pathologic and microbiologic data as well as quality of sample collected [10]. Including an expanded definition that considered cultures from non-sterile sites, deemed here as probable GBS osteomyelitis, nearly doubled the number of cases in our cohort, with few clinical differences between those deemed to have definite and probable GBS osteomyelitis. Even consideration only of cases monomicrobial GBS osteomyelitis, where the pathogenic role of GBS is less questionable, yields an additional 20% cases using the expanded definition. A population-based study that assessed hospitalizations due to invasive and non-invasive GBS infections in Louisville, Kentucky, also confirmed osteomyelitis as a common presentation of GBS infection and found rates of non-invasive disease that were 3–4 times higher than those of invasive disease; that study relied on clinical and microbiological criteria that differ from the ones we applied here [15]. Those observations, together with our findings, suggests that the strict case definitions used to assess invasive GBS disease in population-surveillance studies may overlook a notable proportion of cases of GBS osteomyelitis.

The cases of GBS osteomyelitis identified using the strict case definitions of invasive disease (classified as definite GBS osteomyelitis) had similar demographic and clinical characteristics than cases identified with GBS using our expanded criteria (probable GBS osteomyelitis). As in previous epidemiologic studies of invasive GBS infection, obesity and especially diabetes mellitus were prevalent in this cohort of patients with GBS osteomyelitis [4, 16]. Diabetes mellitus that was not well controlled (HbA1C > 7.5%) occurred more commonly among those with definite GBS osteomyelitis, consistent with previous reports that describe elevated HbA1c as an important risk factor for invasive GBS disease (5). A high proportion of cases also had peripheral vascular disease. Taken together, these findings suggest that diabetic foot infections were contributory to a large proportion of cases with GBS osteomyelitis in this cohort.

The grave outcomes of GBS osteomyelitis in this cohort of US Veterans deserve comment. Short term mortality, even for cases of invasive GBS infection, appeared low. This may be a function of the susceptibility of GBS against most empiric regimens in patients with suspected osteomyelitis. It is notable, however, that 1 in 10 patients with GBS osteomyelitis are dead within a year, which may reflect the burden of comorbidities in this group of patients, and that mortality was similar among patients with definite compared to probable GBS osteomyelitis. In contrast, among cases with GBS osteomyelitis of a lower extremity, those deemed to have definite osteomyelitis were more likely to proceed to an amputation. This difference may result from selection bias, in that patients who proceeded to amputation were also more likely to have a bone specimen available for culture and GBS isolation, thereby fulfilling the criteria for invasive disease.

The large proportion of cases deemed to have probable GBS osteomyelitis that had polymicrobial infection may also reflect differences in the types of samples collected, which included non-sterile sites like wounds and ulcers. For these samples, the microbiology laboratory may choose to only report GBS if it grows in significant quantities, whereas any presence of *S. aureus* and *P. aeruginosa* is typically reported. If this is the case, GBS can likely be considered a pathogen, giving credence to polymicrobial osteomyelitis. Alternatively, the microbiology laboratory may report any presence of GBS regardless of the presence of other pathogens, in which case the pathogenic role of GBS is more difficult to discern. Unfortunately, in this study we were not able to assess the protocols and practices of microbiology laboratories that processed these samples.

Cultures collected from patients with definite osteomyelitis were significantly less likely to have other organisms, including *S. aureus* and *P. aeruginosa.* Nevertheless, these pathogens were found in up to a quarter of cases defined by isolation of GBS from a sterile site (*i.e.,* bone and blood). In this scenario, it is difficult to establish which is the predominant pathogen, as they may display synergistic interactions that enhance their colonization, virulence and persistence [17]. The rates of mortality and of lower extremity amputation among patients with cultures that were monomicrobial for GBS were similar to those for the entire cohort. These results suggests that GBS has as much pathogenic potential as other bacterial species commonly recognized in association with osteomyelitis, such as *S. aureus*. As most antibiotic regimens that treat *S. aureus* osteomyelitis are effective against GBS, the risk of inadvertently selecting inappropriate therapy against GBS is low [18].

Our study has several important limitations. First, the results of this study may not be generalizable to other populations because of the different sociodemographic and health patterns that exist in the VA population. Specifically, VA patients are predominantly white, non-Latinx males with a high burden of chronic medical conditions [19]. Second, the definitions employed in this study were based on clinical and administrative data, including ICD codes, CPT codes for amputation and imaging studies, and identification of the site of the GBS culture; ambiguity in labeling and coding errors may have led to misclassification of some cases. Third, we did not examine the results of imaging studies to confirm the diagnosis of osteomyelitis because radiologic interpretations are not readily available through the CDW, thus potentially overestimating the number of cases of probable GBS osteomyelitis. Fourth, there is potential for misclassification of cases with GBS identified from abscesses that occur in a sterile site or from exposed bone that is no longer sterile, and of cases with discrepancies between blood and bone cultures. Finally, practices to isolate and report GBS in clinical specimens, especially in polymicrobial cultures from non-sterile sites, may not be uniform across different microbiology laboratories and may have changed over the study period. Similarly, this retrospective study did not report the serotypes or the antibiotic susceptibilities of GBS isolates since these are not routinely determined at microbiology laboratories serving VA medical centers.

## Conclusions

Clinical characteristics and outcomes of US Veterans with GBS osteomyelitis were similar in those with probable GBS osteomyelitis (determined by diagnostic codes, imaging codes, and isolation of GBS from relevant non-sterile sites) and those with definite GBS osteomyelitis (determined by diagnostic codes and isolation of GBS from sterile sites). This suggests that cases of probable GBS osteomyelitis, which represent non-invasive GBS infection, are as important in terms of their frequency and clinical impact as definite cases, which are akin to invasive GBS infection. Consequently, population-based surveillance of invasive GBS disease likely underestimates the overall incidence of GBS osteomyelitis. Accurate estimation of the burden of GBS osteomyelitis may help guide preventive efforts among patients at risk, mitigating the overall impact of this type of GBS infection.

## Supplementary Information

**Additional file 1. Supplemental Tables 1-4** detail the International Classofication of Diseases (ICD) and current procedural terminology (CPT) codes as part of case identification.

## Data Availability

The data that support the findings of this study are available on request from the corresponding author [RJ or FP]. The data are not publicly available because they personal identifiable information that could compromise privacy.
