# Supplementary material for: Expanding the definition beyond surveillance criteria reveals a large burden of osteomyelitis caused by group B Streptococcus in the United States Veterans Health Administration
Source: BMC Infect Dis. 2022 Mar 8;22:237. doi: 10.1186/s12879-022-07238-0 (PMC8905819; doi:10.1186/s12879-022-07238-0)
Supplement: Supplementary file 1 — Additional file 1. Supplemental Tables 1-4 detail the International Classofication of Diseases (ICD) and current procedural terminology (CPT) codes as part of case identification. [file 12879_2022_7238_MOESM1_ESM.docx]

**Supplementary Materials**

**Table 1.** International Classification of Diseases (ICD) Codes used to determine osteomyelitis

| ICD9 Codes | 729.4, 728.86, 040.0,376.03, 730.00, 730.01, 730.02, 730.03, 730.04, 730.05, 730.06, 730.08, 730.09, 730.10, 730.11, 730.12, 730.13, 730.14, 730.15, 730.16, 730.18, 730.19, 730.20, 730.21, 730.22, 730.23, 730.24, 730.25, 730.26, 730.28, 730.29, 730.80, 730.81, 730.82, 730.83, 730.84, 730.85, 730.86, 730.88, 730.89, 730.90, 730.91, 730.92, 730.93, 730.94, 730.95, 730.96, 730.98, 730.99, 730.07, 730.17, 730.27, 730.87, 730.97 |
| --- | --- |
| ICD10 Codes | M72.6, N49.3, H05.021, H05.022, H05.023, H05.029, M46.22, M46.23, M46.24, M46.25, M46.26, M46.30, M46.31, M46.32, M46.33, M46.34, M46.35, M46.27, M46.37, M46.38, M46.39, M46.28, M46.36, M46.50, M46.51, M46.52, M46.53, M46.54, M46.55, M46.56, M46.57, M46.58, M46.59, M86.00, M86.011, M86.012, M86.019, M86.021, M86.022, M86.029, M86.031, M86.032, M86.039, M86.041, M86.042, M86.049, M86.051, M86.052, M86.059, M86.061, M86.062, M86.069, M86.08, M86.09, M86.10, M86.111, M86.112, M86.119, M86.121, M86.122, M86.129, M86.131, M86.132, M86.139, M86.141, M86.142, M86.149, M86.151, M86.152, M86.159, M86.161, M86.162, M86.169, M86.18, M86.19, M86.20, M86.211, M86.212, M86.219, M86.221, M86.222, M86.229, M86.231, M86.232, M86.239, M86.241, M86.242, M86.249, M86.251, M86.252, M46.80, M46.81, M86.259, M86.261, M86.262, M86.269, M86.28, M86.29, M86.30, M86.311, M86.312, M86.319, M86.321, M86.322, M86.329, M86.331, M86.332, M86.339, M86.341, M86.342, M86.349, M86.351, M86.352, M86.359, M86.361, M86.362, M86.369, M86.38, M86.39, M86.40, M86.411, M86.412, M86.419, M86.421, M86.422, M86.429, M86.431, M86.432, M86.439, M86.441, M86.442, M86.449, M86.451, M86.452, M86.459, M86.461, M86.462, M86.469, M86.48, M86.49, M86.50, M86.511, M86.512, M86.519, M86.521, M86.522, M86.529, M86.531, M86.532, M86.539, M86.541, M86.542, M86.549, M86.551, M86.552, M86.559, M86.561, M86.562, M86.569, M86.58, M86.59, M86.60, M86.611, M86.612, M86.619, M86.621, M86.622, M86.629, M86.631, M86.632, M86.639, M86.641, M86.642, M86.649, M86.651, M86.652, M86.659, M86.661, M86.662, M86.669, M86.68, M86.69, M86.8X0, M86.8X1, M86.8X2, M86.8X3, M86.8X4, M86.8X5, M86.8X6, M86.8X8, M86.8X9, M86.9, T86.832, M46.20, M46.21, M46.40, M46.41, M46.43, M46.45, M46.42, M46.47, M46.44, M46.49, M46.46, M46.48, M86.671, M86.672, M86.679, M86.8X7, M86.071, M86.072, M86.079, M86.171, M86.172, M86.179, M86.271, M86.272, M86.279, M86.371, M86.372, M86.379, M86.471, M86.472, M86.479, M86.571, M86.572, M86.579 |

**Table 2.** Current procedural terminology (CPT) codes to select patients with imaging study used to assess for osteomyelitis

| CPT codes | 70110, 70130, 70150, 70160, 70200, 70210, 70220, 70260, 70330, 70336, 70480, 70481, 70482, 70487, 70488, 70540, 70543, 71100, 71101, 71110, 71120, 71130, 71550, 71551, 71552, 72040, 72050, 72052, 72070, 72082, 72100, 72114, 72125, 72126, 72128, 72129, 72131, 72132, 72141, 72142, 72146, 72147, 72148, 72149, 72156, 72157, 72158, 72159, 72170, 72192, 72193, 72194, 72195, 72196, 72197, 72200, 72202, 72220, 73000, 73010, 73030, 73050, 73060, 73070, 73090, 73100, 73110, 73120, 73130, 73140, 73200, 73201, 73202, 73218, 73219, 73220, 73221, 73222, 73223, 73501, 73502, 73503, 73521, 73522 ,73523, 73552, 73560, 73562, 73564, 73590, 73600, 73610, 73620, 73630, 73650, 73660, 73700, 73701, 73702, 73718, 73719, 73720, 73721, 73722, 73723, 74185, 76010, 77077, 78306, 78315, 78814, 78815, 78816 |
| --- | --- |

**Table 3.** International Classification of Diseases (ICD) Codes used to determine lower extremity osteomyelitis

| ICD9 Codes | 730.05, 730.06, 730.07, 730.15, 730.16, 730.17, 730.25, 730.26, 730.27, 730.36, 730.37, 730.85, 730.86, 730.87, 730.97 |
| --- | --- |
| ICD10 Codes | M86.372, M86.371, M86.179, M86.662, M86.651, M86.661, M86.8X5, M86.261, M86.472, M86.462, M86.471, M86.461. M86.469, M86.171, M86.161, M86.672, M86.8X7, M86.452, M86.451, M86.459, M86.172, M86.152, M86.162, M86.159, M86.571, M86.659, M86.272, M86.072, M86.052, M86.071, M86.151, M86.572, M86.561, M86.652, M86.671, M86.679, M86.8X6, M86.271 |

**Table 4.** Current procedural terminology (CPT) codes to select patients with lower extremity amputation

| CPT codes | 27880, 27881, 27886, 27888, 27889, 28120, 27882, 27598, 27590 |
| --- | --- |
